# Supplementary material for: The effects of type of recovery in resistance exercise on responses of platelet indices and hemodynamic variables
Source: PLoS One. 2023 Aug 18;18(8):e0290076. doi: 10.1371/journal.pone.0290076 (PMC10437857; doi:10.1371/journal.pone.0290076)
Supplement: S2 File — (DOCX) [file pone.0290076.s002.docx]

Inclusivity in global research

PLOS’ policy on inclusivity in global research aims to improve transparency in the reporting of research performed outside of researchers’ own country or community and ensures that PLOS publications reporting global research adhere to high standards for research ethics and authorship. Authors of relevant research articles may be asked to complete the questionnaire below, which outlines ethical, cultural, and scientific considerations specific to inclusivity in global research. This questionnaire may be requested when researchers have travelled to a different country to conduct research, if research uses samples collected in another country, research with Indigenous populations or their lands, or if research is on cultural artefacts. Researchers travelling to another country solely to use laboratory equipment will not normally be required to complete the questionnaire. However, the questionnaire can be requested at the journal’s discretion for any submission – if you have been requested to complete this questionnaire by the PLOS journal you submitted to, please do so.

Please complete the questionnaire below and include this as a Supporting Information file with your manuscript. Note that if your paper is accepted for publication, this checklist will be published with your article in the supporting information files. Please ensure that you reference the checklist in the main body of your manuscript. We suggest adding a subsection ‘Inclusivity in global research’ to your Methods section and adding the following sentence: “Additional information regarding the ethical, cultural, and scientific considerations specific to inclusivity in global research is included in the Supporting Information (SX Checklist)”

The questions have been designed to be applicable to a wide range of study types, and there are subsections for both human subjects research and non-human subjects research. If any of the questions are not relevant to your research please mark them as “N/A” as appropriate.

**Ethical considerations, permits and authorship**

*This section is applicable to all research types.*

Provide details as to who granted permissions and/or consent for the study to take place in the Methods section of your manuscript. This should include the names of **all** ethics boards, governmental organizations, community leaders or other bodies that provided approval for the study. If individuals provided approval refer to these people by their role or title but do not list their name(s).

Reported on page number: In the method section (page number 6) all the detail is provided.

If there were any deviations from the study protocol after approval was obtained please provide details of these changes in the Methods section of your manuscript.
Did this study involve local collaborators that are residents of the country where the research was conducted or members of the community studied? If you do not have any authors from said communities, please provide an explanation for this below.

Reported on page number: No, there was no deviations from the study protocol after approval was obtained.

Our study was conducted within an academic setting and involved 12 graduate students as participants. As such, our collaboration was primarily with these individuals who are indeed residents of the country where the research was conducted.

As for the authors of the study, they are members of the same academic institution as the participants.

Everyone listed as an author should meet PLOS’ criteria for authorship and all individuals who meet these criteria should be included in the author byline, rather than the acknowledgements. For further information please see the journal’s Authorship Policy.

**Human subjects research (e.g. health research, medical research, cross-cultural psychology)**

Did you obtain written informed consent from a representative of the local community or region before the research took place? How did you establish who speaks for the community? Details of written informed consent obtained from study participants should be reported separately in the Methods section of your manuscript.

For our research study, we adhered strictly to the ethical guidelines and approval processes set out by our university's ethics committee. They reviewed and approved all study procedures, as well as the consent form we utilized for participant involvement.

Regarding the question, "Did you obtain written informed consent from a representative of the local community or region before the research took place?", our study did not involve a specific community or region in a way that required consent beyond individual participants. Rather, our study involved 12 individual human participants, each of whom provided written informed consent after receiving a thorough explanation of the study procedures, potential risks and benefits, confidentiality measures, and their right to withdraw from the study at any point without negative consequences.

As for the question "How did you establish who speaks for the community?", as the nature of our research was focused on individual participants rather than a specific community or region, we did not have to identify a representative or spokesperson for a community. Each of our participants was treated as an autonomous individual, capable of making decisions about their own involvement in the study.

How did members of the local community provide input on the aims of the research investigation, its methodology, and its anticipated outcome(s)?

In our study, we worked with 12 individual human participants, rather than a specific local community. These participants were were recruited through a combination of poster announcements and online advertisements distributed within the university. Therefore, the research design, aims, and methodology were primarily informed by the scientific and ethical guidelines applicable to our field and by our university's ethics committee rather than community input.

However, each participant was fully informed about the aims of the research, the methodology, and the anticipated outcomes prior to their participation. They were given opportunities to ask questions, provide input, and express any concerns they might have had. In this way, we ensured that our participants' perspectives were respected and taken into consideration throughout the research process.

When engaging with the local community, how did you ensure that the informed consent documents and other materials could be understood by local stakeholders?

N/A

Will the findings of the research be made available in an understandable format to stakeholders in the community where the study was conducted (e.g. via a presentation, summary report, copies of publications, etc.)? Please provide details of how this will be achieved.

Yes, we are committed to ensuring that the findings of our research are accessible and understandable to all stakeholders, including our study participants. Given that our study involved a group of 12 graduate students in Exercise Physiology, we understood the importance of providing results in a format that was meaningful and relevant to their academic and professional interests.

Upon the completion of our study, we summarized the results in Persian, the language that all participants are comfortable with, ensuring the findings are accessible and clear. Each participant was given a copy of this summary. We found this to be an effective method for communicating our findings, as all of our participants have a strong background in the topic and can interpret and use the findings in their studies or work.

In terms of wider dissemination, we also plan to publish our findings in an academic journal, which are accessible to the broader scientific community. If relevant and appropriate, we are open to organizing additional presentations or workshops for interested parties, to further share our research outcomes and implications.

**Non-human subjects research using specimens/ animals collected as part of the study, or those housed in archival collections. Examples include archaeology, paleontology, botany and zoology.**

Did the permission you obtained from a local authority to perform the study include an agreement on access to outputs and benefit sharing? This may include procedures to enable fair distribution of the benefits and resources arising from the research performed. Please include any details of Prior Informed Consent and Benefit Sharing Agreements obtained. These may be required by field-specific regulations, for example the Convention on Biological Diversity (CBD) and the associated Nagoya Protocol.

N/A

If the material used in your study was imported, please A) provide the year it was imported and B) indicate whether permits were obtained to import/export the materials used, C) provide details of any permits obtained. If this information is not available, please indicate this.

N/A

If you used archival specimens, please state how the material used in your study was acquired by the institute it is held in and provide details of any permits obtained for the original excavations/ sample collection. If this information is not available, please indicate this.

N/A

How was the potential cultural significance of the materials collected in your study to local communities considered in your research design? Were Indigenous peoples and/or local researchers and institutions involved with archaeological excavations / collection of specimens? If so, please provide a description of their involvement.

N/A

If your manuscript includes photographs of human remains please indicate whether authors obtained permission from descendants or affiliated cultural communities to do so.

N/A
